# Intelligent neonatal monitoring based on a virtual thermal sensor

**DOI:** 10.1186/1471-2342-14-9

**Published:** 2014-03-02

**Authors:** Abbas K Abbas, Steffen Leonhardt

**Affiliations:** 1 Philips Chair for Medical Information Technology (MedIT), RWTH Aachen University, Pauwelsstr. 20, D 52074 Aachen, Germany

**Keywords:** Thermography imaging, Neonatal incubator, Virtual sensor, ROI matching, Thermal signature, ROI tracking

## Abstract

**Background:**

Temperature measurement is a vital part of daily neonatal care. Accurate measurements are important for detecting deviations from normal values for both optimal incubator and radiant warmer functioning. The purpose of monitoring the temperature is to maintain the infant in a thermoneutral environmental zone. This physiological zone is defined as the narrow range of environmental temperatures in which the infant maintains a normal body temperature without increasing his or her metabolic rate and thus oxygen consumption. Although the temperature measurement gold standard is the skin electrode, infrared thermography (IRT) should be considered as an effortless and reliable tool for measuring and mapping human skin temperature distribution and assist in assessing thermoregulatory reflexes.

**Methods:**

Body surface temperature was recorded under several clinical conditions using an infrared thermography imaging technique. Temperature distributions were recorded as real-time video, which was analyzed to evaluate mean skin temperatures. Emissivity variations were considered for optimal neonatal IRT correction for which the compensation vector was overlaid on the tracking algorithm to improve the temperature reading. Finally, a tracking algorithm was designed for active follow-up of the defined region of interest over a neonate’s geometry.

**Results:**

The outcomes obtained from the thermal virtual sensor demonstrate its ability to accurately track different geometric profiles and shapes over the external anatomy of a neonate. Only a small percentage of the motion detection attempts failed to fit tracking scenarios due to the lack of a properly matching matrix for the ROI profile over neonate’s body surface.

**Conclusions:**

This paper presents the design and implementation of a virtual temperature sensing application that can assist neonatologists in interpreting a neonate’s skin temperature patterns. Regarding the surface temperature, the influence of different environmental conditions inside the incubator has been confirming.

## Background

Recently, the rapid improvement in medical thermography technologies in various clinical fields has promoted the use of thermography imaging as a contactless physiological sensor. In particular, neonatal intensive medicine is a clinical field in which infrared thermography may play a future role in non-invasive monitors.

Initially, Clark et al. [1] performed the first clinical trials using direct thermography measurement in neonates, which was dated back to 1980. To perform non-invasive skin temperature measurements, the setup included a hole in the roof of the incubator and the assistance of a mirror system; these additions [1,2] allowed for real-time measurements of thermal reputation.

Adams et al. [3] achieved successful direct thermography imaging in the earliest minutes of life by using a long-wave infrared (LWIR) system. In that project, continuous thermal monitoring of the neonate was accomplished at intermittent intervals ranging between 20 and 30 minutes at the initial stage. Then, a modified protocol was defined to monitor preterm infants inside a convective incubator, kangaroo mother care, and open radiant warmer. The results were compared with values obtained from multiple weighted measurements of resistance temperature device (RTD) sensors.

Pavlidis et al. [4-6] developed a tracking system for infrared thermography as part of an augmented computer vision system. This development was based on a coalitional tracking approach in which a distinct region of interest (ROI) was defined over the neonate’s face and its position was tracked over numerous infant motion planes.

Recently, Abbas et al. [7] developed a concept for non-contact respiration monitoring in infants based on IR thermography (IRT). This technique also tracks the nostrils’ thermal signature to detect the infant’s breathing rate at a distance, and it provides an insightful analysis of possible error sources within the neonatal IRT (NIRT) imaging technique. The need for a robust and intelligent temperature monitoring methodology has increased, which makes NIRT imaging a suitable candidate for contactless temperature measurement and observation inside neonatal intensive care unit (NICU) facilities [2,8].

The NIRT method demonstrates good outcomes for the real-time and continuous quantification of a neonate’s surface and core temperatures; however, it lacks the ability to estimate the real temperature value on a neonate’s body surface accurately. This lack of reliability is mainly due to the unknown emissivity, ε. For reference, the experimenter could utilize an emissivity value ε for a known material surface or could utilize fabric supplies, such as a hand band or head caps, sutured with a material of known emissivity, such as copper, polished steel, or polyvinyle-flouride electrical tape (e.g., scotch-764). However, in such clinical study, it is impossible to use like material due to the hygienic and disinfection concern that roses within the utilization of these material inside infant incubators.

### Thermal imaging

Radiation in the long wave infrared (LWIR) bands (8-14 μm) is important because the human body emits most of its thermal radiation, which encodes valuable physiologic information, in this region of electromagnetic spectrum. This vital information, if properly processed and analyzed, may be used in many biomedical applications, such as mean body temperature mapping and arterial pulse measurements [6,9,10]. A solid base that includes an understanding of the physics of image formation principles, the choice of imaging IR band, and instrumentation is crucial for successful biometrics signature processing. Such signatures include superficial vessel blood flow [11], forehead mean temperature, and nostril thermal patterns [4,12-14].

**Figure 1:**
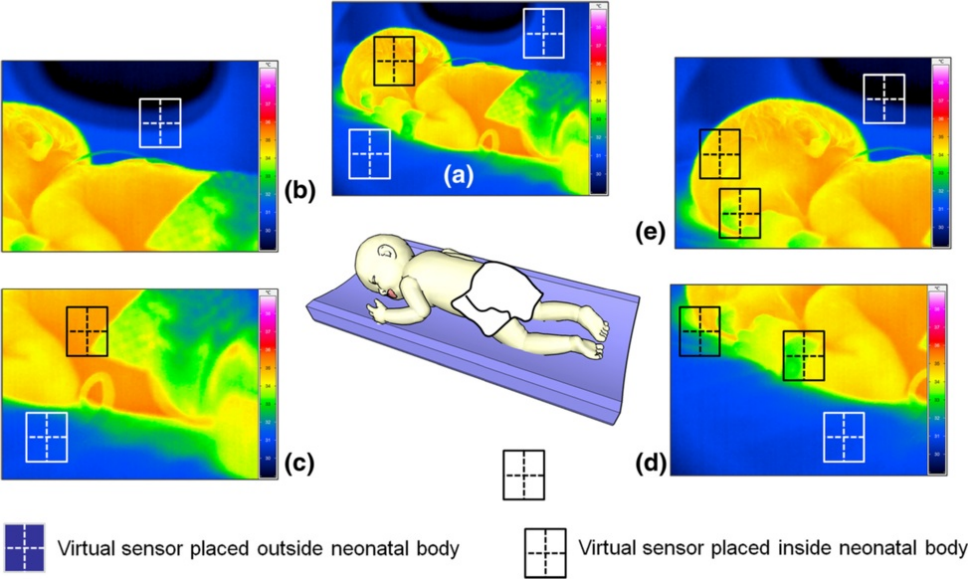
**Positions of different possible locations of the virtual temperature sensor developed for NIRT.** Directing from **(a)** initial position of black window (on face) as reference sensor and white windows as ancillary points showing spatial variation over **(b, c, d and e)** to register different temperature of the neonate and incubator.

Possible IRT tracking and monitoring sites on a neonate’s body are displayed in Figure 1; these spatial points will be the reference sites for virtual temperature sensing as the issue is discussed further in this paper.

Thermography imaging offers a high-quality concept for the observation and monitoring of different physiological processes [8,15,16]. Recently, we used IR thermal imaging to monitor and map the temperature distribution over the preterm infant’s body [12,17,18]. We believe that this technique will become an alternative technique in the future to gold-standard technologies in neonatal temperature monitoring and control [19].

## Methods

**Figure 2:**
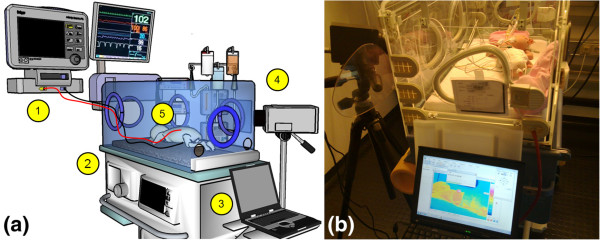
**Experimental setup of the NIRT Clinical study by using thermography imaging technique in association with a clinical temperature measurement (a) 3D schematic for components and elements of the setup (1) patient monitoring system, (2) convective infant incubator unit, (3) analysis workstation, (4) IR camera and (5) infant with two skin electrodes connected. (b)** Photograph of typical clinical setting.

All measurements were performed using a VarioCAM® hr head (InfraTec GmbH, Germany) IR camera (LWIR, 7 μm to 14 μm). The camera transferred the thermal map to a PC via the IEEE 1394 FireWire interface. The neonate’s thermal images were taken inside a convective infant incubator (Caleo, Draeger AG, Germany) and converted to a 2D array containing temperature information within the LabVIEW software platform. Additionally, these data were used to test the algorithm software’s ability to track the specified virtual temperature sensor points on a neonate’s skin after motion. Figure 2 illustrates a typical setting for NIRT clinical study inside a convective incubator.

### Thermography imaging experiment design

Only ten newborn infants were selected to participate in the clinical study, five of them were under radiant warmer therapy and the rest are placed inside convective incubator. A referential ground truth measurement was implemented by using skin temperature electrodes as gold standards. The accuracy of these clinical skin electrodes is (± 0.1°C). The NIRT imaging and measurement was performed at the Department of Neonatology (RWTH Aachen University Hospital), and this has been approved by the medical ethics committee of the RWTH Aachen University Hospital, issued on 19 August 2009 with reference code (EK032/09). The acquired thermography datasets used for testing the tracking algorithm. Each dataset contained one measurement scene consisting of a newborn infant undergoing thermography inside a convective incubator or under a radiant warmer. The tracking time was approximately 20 minutes for each subject with a frame rate of 25 fps, and the measurements were conducted as a real-time imaging operation. In principle, a higher frame rate (up to 50 fps) could be achieved; however, a higher frame rate would increase the size of the thermography data to an out-of-memory level in many PCs.

**Figure 3:**
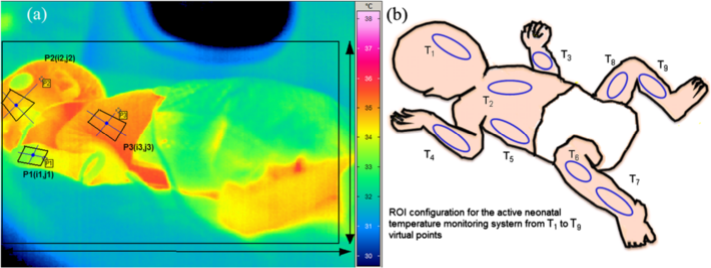
Virtual geometric profiles utilized in ROI tracking for NIRT images (a) and the corresponding profiles over the neonate’s body (b), which will be tracked throughout all of the video frames (the perspective of the overhead ROI changes).

Principally, the selected thermography datasets often included involuntary movements of the neonate during the 20 minutes of thermography acquisition time. The thermography data featured out-of-plane rotation of the facial tissue, hands, feet, and main trunk as the neonates rotated their heads left, right, up, down, or in a random motion. For covering all planes and geometry of the neonate, we configure and selected ROI over the neonate’s skin to guarantee effective temperature detection over examination time (Figure 3).

**Figure 4:**
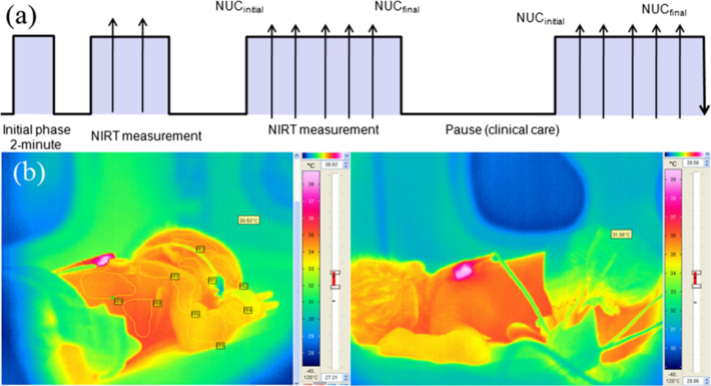
**NIRT protocol used in the virtual sensor tracking. (a)** ROI profiles located over the neonate’s skin **(b)** and an alternative layout of the neonate prior to NIRT imaging.

A ring-projection transformation was selected in the tracker hierarchy to be compared against the active ROI tracker. The calibration phase of the IR camera was performed directly throughout the measurement time. The typical NIRT protocol sequence used in this study explained in Figure 4, in which the NIRT measurement phase indicates different intervals throughout time.

### IR thermal camera calibration setting

The calibration process of the thermal camera took place inside the NICU ward in synchronization with the NIRT measurement phases [20]. This process is called automatic non-uniformity calibration (ANUC), and the procedure compensates for temperature drift during measurements. In addition, the selected field of view (FOV) for the camera assured that there is no influence on thermography resolution during NIRT imaging despite the inclined side angle of the thermal camera within the allocated FOV. This was confirmed during analysis and modeling of heat fluxes dissipated from neonate within NIRT measurement [19].

Temperature and humidity variations inside the convective incubator are commonly considered the main factors that prevent accurate temperature calibration. Therefore, to avoid any incorrect temperature registration and physical related errors in NIRT imaging, the calibration process was implemented during the clinical measurement using the IRBIS® Professional software of the IR camera. Objects of interest (OOIs) inside the acquired thermogram were selected and the environmental, incubator and object settings were performed through an IR transparent window (with 0.01 mm thickness) made of polyethylene (PE) material [3].

The transmission of IR radiation through the foil is between 0.92 and 0.94. Therefore, this transparent foil was chosen to block the opened incubator clapper while allowing the baby inside the incubator to be visualized because the Plexiglas® material of the incubator hood is an IR-reflecting material with emissivity values reaching 0.97 [1,21].

**Figure 5:**
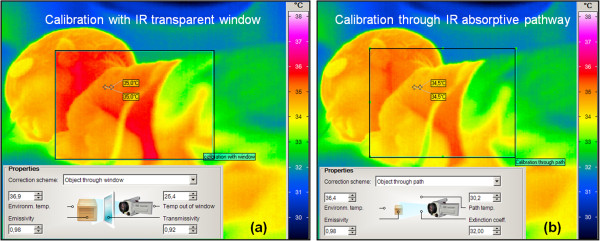
Two thermograms showing the effect of geometric correction of the neonate, it is noticed that the neonates skin temperature in thermogram (a) with higher value than in thermogram (b) of the same neonate, therefore, correction step prior to NIRT imaging is important for accurate thermography acquisition.

A geometric correction was applied to the acquired thermography using selected region of interests (ROI) over the neonate’s skin and setting the physical parameters (e.g., incubator air temperature, outside window temperature, humidity, IR transmission of PE thin-foil and body temperature) for optimal thermography correction. Figure 5 shows the difference in calibration setting between different thermography scenes where in scene (a) the thermography imaging performed through IR-transparent window and in scene (b) thermography imaging performed directly without interfering media [19].

Moreover, the data were registered against an emissivity equal to unity (considering neonatal skin as a typical blackbody radiator), although the actual value of emissivity was equal to 0.972 [22,23]. This correction strategy plays a vital role in accurate temperature mapping because any slight difference in the emissivity value will tend to add inaccuracies to the temperature reading from the IR camera.

### Thermal virtual sensor architecture

**Figure 6:**
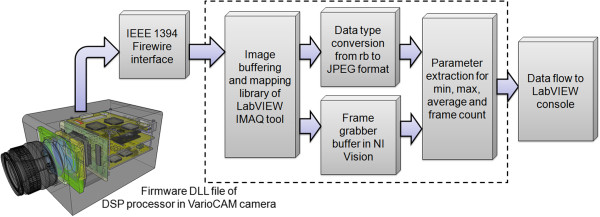
Architecture of thermography imaging acquisition within the LabVIEW platform for the virtual thermal sensor.

The term “Virtual InfraRed SENSor” (VIRSENS) relates to a sensing method based on augmented visual or physical measurements. In this work, a virtual temperature sensor was developed wherein contactless temperature measurements essentially replace the clinical gold standards. Furthermore, virtual sensor tracking software was developed using LabVIEW® Vision Assistance (National Instruments®) as an integrated toolkit. This software allowed the thermal camera to be connected directly the LabVIEW console by using a native interface file provided by the manufacturer (Figure 6).

**Figure 7:**
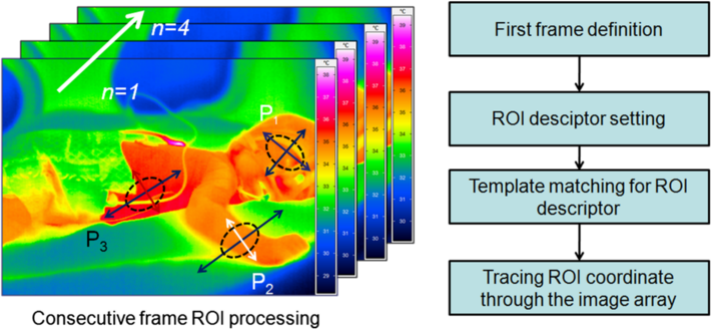
(Left): Successive thermography frame-by-frame definition and tracking of selected ROIs from the different body parts of a neonate, (Right): Simplified flow diagram for tracking method in the virtual sensor.

Thermography acquisitions began after IR camera calibration and were followed by the extraction of the thermal data from the color space of the image; this task formed a crucial step of the VIRSENS concept. Moreover, the selection of the ROI array was initiated afterward to set the tracking coordinates of the neonate’s body regions to be implemented the image-processing loop and architecture (Figure 7).

### Tracking technique

**Figure 8:**
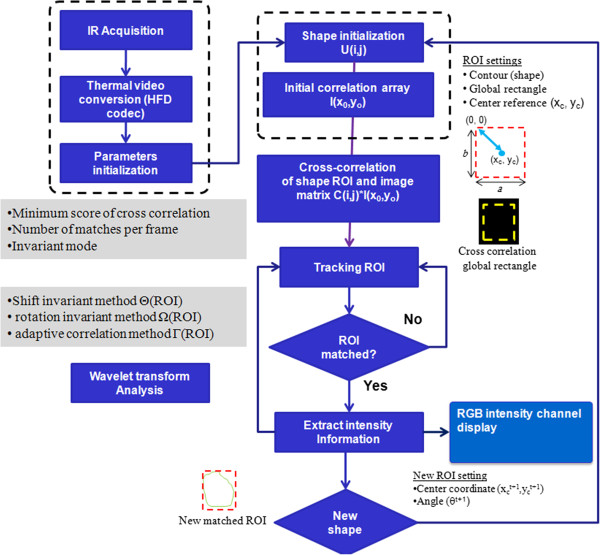
Fundamental steps of the ROI tracking algorithm for NIRT virtual thermal sensing, illustrating processing flow from thermal acquisition down to the surface temperature presentation.

The key aspect for robust virtual sensing is the tracking method, which should accurately monitor the motion of the target surface even in the presence of partial occlusion or deformation [24]. This tracking system is applied to follow the motion of the target’s outline (and not only superficial features) [25-28]. Generally, motion tracking is not a straight forward process; it depends on the proper definition of the tracked anatomical geometry and the ability to follow-up and mark the defined ROI over multiple thermography frames (Figures 7 and 8).

**Figure 9:**
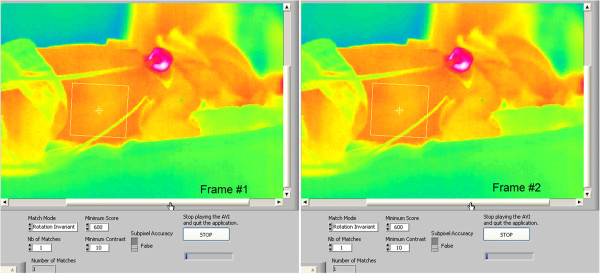
Two successive ROI tracking images used in the virtual sensing technique, were the ROI profile moves due to the neonate’s body movements along relative coordinates.

Primarily, the tracking algorithm can be divided into five main stages, as illustrated in Figure 8: IR thermography acquisition, ROI geometry profile definition, object coordinate tracking, information extraction, and sensor display. The manner in which the active ROI moves through the image frames is illustrated in Figure 9, where the yellow rectangle moves with the relative motion of the baby inside the camera’s field of view (FOV).

When template matching, the ring projection template (RPT) process was used to address rotational variations within the thermography-imaging scene. The RPT reduces a 2D thermogram image into a 1D vector. In general, this task is used as a pre-processing step in the VIRSENS approach.

We define the initial template to be *T(x,y)* of size *(M* × *N).* The RPT process begins by deriving a center point on the Template *T(x,y)* that is denoted as *(x*_*c*_*,y*_*c*_*)*. Subsequently, the Cartesian frame coordinate Template *T(x,y)* is transformed into polar frame coordinates based on the following relations:

x = r cosθ (for horizontal reference) , y = r sinθ (for vertical reference)

where

(1) $$r=\left(\;,\mathrm{int}\right)\left(\sqrt{{\left(x-x_{c}\right)}^{2}-{\left(y-y_{c}\right)}^{2}}\right),r\in \left[0,R\right],R=\min\left(M,N\right)$$

Basically, the ring projection in the selected template *T(x,y)* at radius *r* is denoted as *P*_*T*_*(r)* and is defined as follows:

(2) $$P_{T}\left(r\right)=\frac{1}{S_{r}}\sum_{k}T\left(r\cos\theta_{k},r\sin\theta_{k}\right),$$

where *S*_*r*_ is the total number of pixels falling on the circle of radius *r = 0,1,2,…,R* and *k* denotes the number of correlation iterations in template matching kernel. Note that *P*_*T*_*(r)* is defined as the mean pixel intensity along a circle whose radius to the center of the template has equal order in the correlation computation.

Because the RPT is synthesized along circular rings of increasing radii, the derived 1D RPT on the thermography image is invariant to the rotation of its corresponding 2D image template. To effectively obtain the RPT computation along concentric circles, the method employs a look-up table (LUT) whose diameter is set to the size of the template in the ring projection process.

Finally, the RPT is obtained simply by summing up the pixel values along a concentric circle within the template results. For the matching process, the normalized correlation (NC) is adopted in the similarity measurement. Therefore, we consider the following:

(3) $${\vec{P}}_{T}\overset{\Delta }{=}\;\left[P_{T}\left(0\right),P_{T}\left(1\right),\ldots ,P_{T}\left(R\right)\right]$$

and

(4) $${\vec{P}}_{S}\overset{\Delta }{=}\;\left[P_{S}\left(0\right),P_{S}\left(1\right),\ldots ,P_{S}\left(R\right)\right]$$

Generally, the representations of the reference template ring-projection vectors (${\vec{P}}_{T}$) and thermography scene subimage (${\vec{P}}_{S}$) are computed consecutively. The normalization correction (NC) process between the ring projection vectors ${\vec{P}}_{T}$ and ${\vec{P}}_{S}$, denoted by $\langle {\vec{P}}_{T},{\vec{P}}_{S}\rangle$, is defined as

(5) $$\langle {\vec{P}}_{T},{\vec{P}}_{S}\rangle =\;\frac{{\left(\left(R+1\right)\sum_{r=0}^{R}P_{T}\left(r\right)P_{S}\left(r\right)-\sum_{r=0}^{R}P_{T}\left(r\right)\sum_{r=0}^{R}P_{S}\left(r\right)\right)}^{2}\times 100}{\left(\left(R+1\right)\sum_{r=0}^{R}P_{T}{\left(r\right)}^{2}-(\sum_{r=0}^{R}P_{T}\left(r\right)){}^{2}\right)\;\left(\left(R+1\right)\sum_{r=0}^{R}P_{S}{\left(r\right)}^{2}-(\sum_{r=0}^{R}P_{S}\left(r\right)){}^{2}\right)}$$

With this definition, the value is unaffected by rotational and linear changes (at constant gain and contrast offset in the thermal imaging) in the reference template and thermography scene subimage. In addition, the dimensional length of the ring projection vector is only (R + 1). This significantly increases the computational efficiency for the vector

$$\langle {\vec{P}}_{T},{\vec{P}}_{S}\rangle .$$

### Parametric vector approach for template matching

The method proposed here is inspired by the PT method, which is characterized by a decrease in computational complexity when the thermography image involves a change of scale and rotation. Therefore, it is considered a robust solution for the large-scale image data generated in medical thermography [26,29,30]. To obtain rotation/scale invariance in the matching process, a simple approach using a P_T_ vector (template image) and a P_S_ vector (scene subimage) was proposed.

In the VIRSENSE approach, a PT vector ${\vec{P}}_{T_{P}}$ was constructed from a base-ring projection set (${\vec{P}}_{t_{0}},{\vec{P}}_{t_{1}},\ldots ,{\vec{P}}_{t_{N}}$) consisting of the RPTs and including the template image and differently scaled images as follows:

(6) $${\vec{P}}_{T_{P}}\overset{\Delta }{=}\;\frac{{\vec{P}}_{t_{0}}\omega_{0}+{\vec{P}}_{t_{1}}\omega_{1}+\ldots +{\vec{P}}_{t_{N}}\omega_{N}}{\left|{\vec{P}}_{t_{0}}\omega_{0}+{\vec{P}}_{t_{1}}\omega_{1}+\ldots +{\vec{P}}_{t_{N}}\omega_{N}\right|},0.0\leq \omega_{i}\leq 1.0,\sum_{i=0}^{N}\omega_{i}=1.$$

The NC between the scene subimage vector ${\vec{P}}_{S}$ and a *PT* vector ${\vec{P}}_{T_{P}}$ becomes $\langle {\vec{P}}_{T},{\vec{P}}_{S}\rangle$; then, the problem under consideration can be solved by constrained optimization, that is,

(7) $$\max_{\left\{\vec{\omega }\right\}}\langle {\vec{P}}_{T},{\vec{P}}_{S}\rangle ,\mathrm{subject}\;\mathrm{to}\;\sum_{i=0}^{N}\omega_{i}=1.$$

Essentially, the Lagrangian multiplier (LM) method can solve this problem of difference optimization. The solution of $\vec{\omega }$ is given by

(8) $$\vec{\omega }=\frac{L^{-1}\vec{F}}{\left(\vec{n}•L^{-1}\vec{F}\right)},$$

where

$$\begin{array}{l} \vec{\omega }\;\overset{\Delta }{=}\;\left[\begin{array}{c} \omega_{0}\\ \vdots \\ \omega_{N} \end{array}\right],\;L\;\overset{\Delta }{=}\;\left[\begin{array}{ccc} \langle {\vec{P}}_{t_{0}},{\vec{P}}_{t_{0}}\rangle & \ldots & \langle {\vec{P}}_{t_{0}},{\vec{P}}_{t_{N}}\rangle \\ \vdots & \ddots & \vdots \\ \langle {\vec{P}}_{t_{N}},{\vec{P}}_{t_{0}}\rangle & \cdots & \langle {\vec{P}}_{t_{N}},{\vec{P}}_{t_{N}}\rangle \end{array}\right],\\ \;\vec{F}=\left[\begin{array}{c} \langle {\vec{P}}_{S},{\vec{P}}_{t_{0}}\rangle \\ \vdots \\ \langle {\vec{P}}_{S},{\vec{P}}_{t_{N}}\rangle \end{array}\right]\mathrm{and}\;\vec{n}\overset{\Delta }{=}\;\left[\begin{array}{c} 1\\ \vdots \\ 1 \end{array}\right] \end{array}$$

The next step of the algorithm is producing the scaling value *sq* estimation of the scene subimage, which initiates in terms of the following equation

(9) $$\mathrm{sq}=\sum_{i=0}^{N}\omega_{i}s_{i},$$

where *s*_*i*_ for 0 ≤ i ≤ N denotes the different scaling values generated by scaling the template image. The approach enables fast matching in the ROI tracking algorithm. The computational efficiency is significantly increased because the RPT process reduces a 2D thermography image array into a 1D vector. Additionally, the correlation matrix (*L)* can be determined in the training phase while the optimal parameters $\vec{\omega }$, the scaling value obtained directly from the correlation vector $\vec{F}$, and the correlation matrix *L* are determined in the matching phase [30]. In fact, there is no iteration step involved in this tracking template-matching-based algorithm. Therefore, the computational time is considerably reduced.

Generally, this data description is appended to the input template image. During the matching phase, the template descriptor (the ROI descriptor, *P*_*ROI*_*(x*_*T*_*, y*_*T*_*)*) is extracted from the template image and used to search the template in the inspection image [31-33].

The mathematical process of image cross-correlation is simple; the RPT is overlaid on the source thermogram image, and the intensity values for each corresponding pixel are multiplied individually. Additionally, all of the matched templates are summed to produce a single correlation value [32,33].

The correlation value matrix is then scanned for its peak value. This position generally conforms to the position in the source image that most closely matches the template [22,34,35]:

(10) $${\vec{P}}_{T}\equiv {\left[P_{T}\left(0\right),P_{T}\left(1\right),\ldots ,P_{T}\left(R\right)\right]}^{T}.P\left(x,y\right)$$

where *P(x,y)* is the reference template position on the thermography image. The correlation matrix can include several high values that correspond to several instances (events) of tracked templates in the source thermography image [36-38].

### Scale (shift)-and rotation-invariant technique

One of the greatest flaws in cross-correlation is its inability to match objects in a source image that are either a different size or rotated compared to the reference template. These two template-matching mechanisms are used in the ROI descriptor tracking (corresponding to the projected template) in the frame matrix. The mathematical approximation of such a template inside a rectangular contour with *T*_*k*_*(x*_*k*_*,y*_*k*_*)* is as follows:

(11) $$P_{T}\left(u\right)=\frac{1}{S_{k}}\sum_{k}T\left(x_{k},y_{k}\right),$$

To overcome and compensate for this issue throughout the NIRT data frames, the template must be rescanned over the thermography scene image using different rotations and sizes (variances in both the x- and y-axes). This process can be extremely time consuming; consider performing a cross-correlation 360 times just to perform a rotation-invariant match without even sub-degree precision [35,39,40].

**Figure 10:**
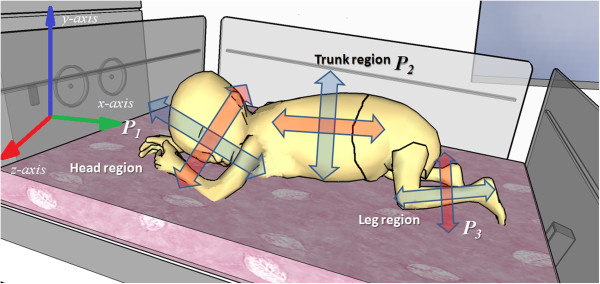
Imaging-plane layout of ROI tracking over a neonate’s different body regions displaying the out-of-plane rotation coordinates that were used to develop an ROI tracking algorithm for medical IRT.

If the tracked portion always has the similar size and no spatial distortion exists, then the virtual sensor does not scan for size variations [4,26,27,41]. The identical principle is applicable for rotation variance if the body part will be repeatedly positioned at the same orientation (Figure 10). In that case, the source thermography image is rescanned using a range of different angles (cross-correlation can typically detect object rotations of approximately ±5° without rescanning) is not necessary.

The detection of object rotations can be accomplished at up to ±12°-18° angle of rotation without rescanning and initializing the reference ROI template. However, the inability of cross-correlation to match objects in a source image that are either a different size or rotated compared to the template is still one of the shortcomings in the rotation-and shift (scale)-invariant method for the object detection system [26,30,42-44].

## Results and discussion

In summary, the results obtained from the virtual sensor demonstrate its ability to accurately track different geometric profiles over the external anatomy of a neonate. Only a small percentage of the motion detection trials failed to track due to the lack of a properly matching matrix for the ROI descriptor under study (see Table 1).

**Table 1:** **Comparison of scoring rate success for VIRSENS in NIRT imaging**^**1**^

**Frame no.**	**Success rate (%)**	**Data-over flow time (ms)**	**Tracked anatomical region**	***p*** **= error rate**	**Correlation coeff.**
**1**	82	1,200	Face-hand/belly	0.0037	0.235
**2**	74	1,403	Face-hand/belly	0.0022	0.171
**3**	80	1,227	Face-hand	0.0015	0.217
**4**	79	1,296	Face-hand/belly	0.0023	0.182
**5**	85	1,372	Face-hand	0.0012	0.302
**6**	86	1,214	Face-hand/belly	0.0031	0.319
**7**	87.2	1,306	Face/hand	0.0024	0.479
**8**	82	1,278	Face/hand/belly	0.0027	0.466
**9**	89.02	1,282	Face-hand/belly	0.0018	0.502
**10**	88.5	1,307	Face/hand	0.0023	0.412

The table also illustrates the correlation of the tracked ROI descriptor over the measurement scene with respect to a newly chosen position of the ROI descriptor.

^1^The table presents the comparison of different success rates for the virtual temperature sensor used within the NIRT imaging for illustrating the scoring percentage of fitted and tracked ROI over the misallocated ones.

The main clinical application of the presented virtual sensor approach is the continuous monitoring of patients without loss of the ROI due to unexpected movements or involuntary motions initiated by the patient. The VIRSENS approach offers the flexibility to perform stress-test infrared thermography, e.g., on treadmills, or to monitor unconscious patients (e.g., under intensive or critical care). Furthermore, this non-contact temperature monitor may become a tool in high-risk missions, such as for pilots or submarine staff [9,12,45], to provide online monitoring of respiration activity through convective heat-loss during expiration and inspiration [7,20,41,46].

To further advance the use of VIRSENS in neonatal medicine, we used embedded contactless temperature monitoring and regulation in a neonatal incubator closed-loop control system. This approach can reduce the need for skin temperature electrodes and the problems associated with their use, such as sensor dislocation, motion artifacts, calibration drift, wire crowding, false connections, and the possibility of infection for newborn infants.

Moreover, this tracking method requires additional validation tests and clinical trials to provide beside the proof-of-concept (POC) of this technology feasibility in the neonatal monitoring field.

**Figure 11:**
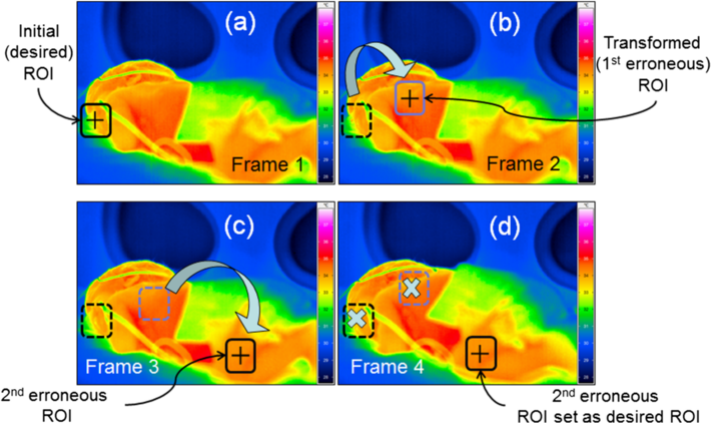
Setting of erroneous ROI tracking over neonate's body regions, displaying the desired ROI position set in sub-figure (a) and changing its position as illustrated in sub-figure (b) through sub-figure (d) by misallocation of the coordinate in the tracking software.

In addition, the ability of VIRSENS to perform geometric identification of selected body parts (e.g., face, hands, legs, interscapular, and maxillary region) (see Additional files 1, 2 and 3) adds a crucial role in anatomical posture identification for neurological reflexes and postural control of neonates. Because the VIRSENS has several misallocated ROI over the neonate’s geometry during the tracking process (Figure 11), which indicates that this method need further optimization and feasibility studies. This believed to be solved when more stable and precise tracking algorithms used in the VIRSENS architecture to become more stable monitoring technique.

Table 2 provides some of quantitative analysis for performance measuring in different thermography datasets within NIRT study. This table showing the scoring of matches of tracked ROI per anatomical regions for seven infants participating in the study. As we can see from Table 2 that the higher success rate of this scoring occurs, in the facial, plane where there is a prominent landmark such as nose, orbital, forehead and maxillofacial regions. Therefore, this is highly discriminated from other anatomy such as hand, arms, legs and trunk can be use the facial tracking as referential template for tracking accuracy and validation procedure of virtual thermal sensor.

**Table 2:** **Comparison of different desired ROI locations of virtual temperature sensor**^**2**^

**NIRT datasets/infant**	**Tracked regions**	**Total desired ROI/region**	**Desired ROI (fitting and tracked)**	**False ROI (misallocated)**	**Scoring percentage %**
**Infant 1**	Facial	4	3	1	75
	Abdominal	6	4	2	66.6
	Upper limb	4	3	1	60
	Lower limb	5	5	0	100
**Infant 2 (radiant warmer)**	Facial	4	2	2	50
	Abdominal	6	4	2	66.6
	Upper limb	5	4	1	75
	Lower limb	5	4	1	75
**Infant 3 (radiant warmer)**	Facial	4	4	0	100
	Abdominal	6	5	1	83.3
	Upper limb	5	5	0	100
	Lower limb	5	4	1	80
**Infant 4 (radiant warmer)**	Facial	4	3	1	75
	Abdominal	6	5	1	83.3
	Upper limb	5	4	1	80
	Lower limb	5	3	2	60
**Infant 5**	Facial	4	3	1	75
	Abdominal	6	4	2	66.6
	Upper limb	5	3	2	60
	Lower limb	5	4	1	80
**Infant 6**	Facial	4	4	0	100
	Abdominal	5	5	0	83.3
	Upper limb	5	3	2	60
	Lower limb	5	3	2	60
**Infant 7**	Facial	4	2	2	50
	Abdominal	6	3	3	50
	Upper limb	5	4	1	80
	Lower limb	5	3	2	60

^2^This table gives the quantitative index for the total numbers of fitted ROIs and missed ROIs over the total number of these selected ROIs for different spatial positions over neonate’s body.

## Conclusion

In this study, a thermal imaging tracking method was proposed and tested based on a template-matching algorithm. The developed method uses a spatially trained ROI tracker whose interactions are modeled using cross-correlations of the ROI template and a searchable IR image. The method’s output provides pixel-level tracking accuracy even in the presence of multidimensional target transformation. The proposed tracking method was effectively tested in thermal and visual datasets featuring facial regions and other anatomical objects.

The thermography tracking system for neonatal monitoring was implemented and tested for clinical monitoring inside NICU unit. The main conclusion from this experiment is that the tracking can be robust over well-calibrated thermography frames and for lesser jerky movements of the neonate. In fact, thermography measurements performed at a distance are beneficial from a psychological viewpoint for both staff and the patient’s relatives but produce challenges from the medical perspective. The tracking problem, which is pivotal in this study, was particularly challenging due to the functional nature of thermal IR imaging and its application in real-time operation.

Moreover, NIRT imaging depicts physiological changes; therefore, it is highly dynamic, non-linear, unpredictable in its uncertainties, and difficult to model. In addition, the estimation of the emissivity value at certain tracking points requires further optimization and development before it can be included in prospective NIRT applications, such as the detection of respiration signatures with the IRTR method or evaluation of superficial blood perfusion over active metabolic regions (e.g., liver and brain). Because these applications would appear to be difficult tasks due to the slow hemodynamic activities of the superficial vessels, the method requires further development and improvement for clinical convention in contactless blood perfusion and hemodynamics parameters.

Furthermore, this physiological tracking application based on thermography might consider a good candidate for running on smartphones and other mobile communication devices. These applications can be a part of the widespread adoption and use of mobile and computing vision technologies is opening new and innovative ways to improve health care delivery. This in turn can transform a mobile platform into a regulated medical monitoring system.

## Statement of consent

An oral consent was gained from the parents of the patent for publication of optical and thermography images and their related files according to medical ethics approval from Medical Ethics committee of the RWTH Aachen University Hospital, issued on 19 August 2009 (EK032/09).

## Abbreviations

Abbreviation: Explanation; IR: Infrared; VIRSENS: Virtual InfraRed SENSor; FOV: Field of view; RPT: Ring projection template; NICU: Neonatal intensive care unit; ROI: Region of interest; OOI: Object of interest; NIRT: Neonatal infrared thermography; PT: Projection template; IRTR: Infrared thermography respiration signal; fps: Frame per second.

## Competing interests

The authors declare that there are no competing interests.

## Authors’ contributions

AK writes the whole article conducting all experimental, technical and analytic works. SL reviews the whole paper and supervises the completely experimental and analytic works. Both authors read and approved the final manuscript.

## Authors’ information

A. K. Abbas was born in Baghdad, Iraq, on Feb. 7^th^, 1979. He received M.Sc. degree in Biomedical engineering in 2004 from Nahrain University, Baghdad, Iraq. He is currently working toward Ph.D. degree (Dr.rer.medic) in the Philips Chair of Medical Information Technology at RWTH Aachen University. His research interests include of Neonatal Infrared Thermography (NIRT) Imaging and developing intelligent monitoring solution for neonatal intensive care units.

S. Leonhardt was born in Frankfurt, Germany, on Nov. 6^th^, 1961. He holds a M.S. in Computer Engineering from SUNY at Buffalo, NY, USA, a Dipl.-Ing. and a Dr.-Ing. degree in Control Engineering from Technical University of Darmstadt, Germany, and a Dr. med. degree from the Medical School of Goethe University, Frankfurt, Germany. He has 5 years of R&D management experience in medical engineering industry and was appointed Head of the Philips Chair of Medical Information Technology at RWTH Aachen University, Aachen, Germany, in 2003. His research interests include physiological measurement techniques, personal health care systems and feedback control systems in medicine.

## Pre-publication history

The pre-publication history for this paper can be accessed here:

http://www.biomedcentral.com/1471-2342/14/9/prepub

## Supplementary Material

Additional file 1 Thermography video file for virtual temperature sensor (VIRSENS) used in NIRT imaging application (respiratory monitoring) for a neonate one cared in open radiant warmer. Click here for file

Additional file 2 Thermography video file for virtual temperature sensor (VIRSENS) used in NIRT imaging application (respiratory monitoring) for a neonate two cared in open radiant warmer. Click here for file

Additional file 3 Thermography video file for virtual temperature sensor (VIRSENS) used in NIRT imaging application (respiratory monitoring) for a neonate three cared in open radiant warmer. Click here for file
